# Artificial Intelligence in Rheumatology: From Algorithms to Clinical Impact in Osteoporosis and Chronic Inflammatory Rheumatic Diseases

**DOI:** 10.3390/jcm15020491

**Published:** 2026-01-08

**Authors:** Marie Doussiere, Ahlem Aboud, Gilles Dequen, Vincent Goëb

**Affiliations:** 1 Department of Rheumatology, University Hospital of Amiens, 80054 Amiens, France; goeb.vincent@chu-amiens.fr; 2 Laboratoire Modélisation, Information, Systèmes (MIS), University of Picardie Jules Verne, 80000 Amiens, France; ahlem.aboud@u-picardie.fr (A.A.); gilles.dequen@u-picardie.fr (G.D.)

**Keywords:** rheumatology, machine learning, artificial intelligence, osteoporosis, rheumatoid arthritis, spondyloarthritis, psoriatic arthritis, rheumatic disease

## Abstract

**Background**: Artificial intelligence (AI) is transforming medicine by supporting data-driven diagnosis, prognosis, and personalized care. In rheumatology, AI applications are rapidly expanding in imaging, disease monitoring, and therapeutic decision support. This review aimed to summarize current evidence on AI in osteoporosis and chronic inflammatory rheumatic diseases, with a focus on methodological robustness and clinical applicability. **Methods**: A narrative review was conducted following SANRA criteria. PubMed and the Cochrane Library were systematically searched for studies published between January 2015 and July 2025 using MeSH terms and free-text keywords related to AI, osteoporosis, and inflammatory rheumatic diseases. A total of 323 articles were included. **Results**: Machine learning and deep learning models show strong performance in osteoporosis for predicting bone mineral density (BMD), bone loss, and fractures. In chronic inflammatory rheumatic diseases, AI improves imaging interpretation, particularly for sacroiliitis. AI tools also demonstrate potential for predicting disease risk and activity, diagnostic support and treatment response. Hybrid models combining imaging, clinical, and biological data appear particularly promising. However, most studies rely on retrospective single-center datasets, with limited external validation, suboptimal explainability, and scarce evidence of real-world implementation. **Conclusions**: AI holds significant promise for advancing diagnosis and personalized management in osteoporosis and rheumatic diseases. However, major challenges persist, including heterogeneous data quality, inconsistent methodological reporting, limited clinical validation, and barriers to integration into routine practice. Bridging the gap between algorithmic performance and clinical impact will require prospective studies, robust validation frameworks, and strategies to build trust among clinicians and patients.

## 1. Introduction

Artificial intelligence (AI) is transforming healthcare by enabling advanced data analysis, predictive modeling, and decision support. Rheumatology offers a high potential for such applications, as chronic inflammatory rheumatic diseases and osteoporosis represent major clinical challenges with important diagnostic and therapeutic needs. AI may support earlier diagnosis, individualized prognostic assessment, and optimized treatment strategies in these conditions. The aim of this systematic review is to synthesize current and emerging applications of AI in rheumatology, with a particular focus on osteoporosis and chronic inflammatory rheumatic diseases, including rheumatoid arthritis (RA), spondyloarthritis (SpA) and psoriatic arthritis (PsA).

## 2. Relevant Sections

### 2.1. Review Methodology

This narrative review was conducted in line with the SANRA quality criteria for narrative reviews, applying a systematic search strategy to enhance comprehensiveness. We searched PubMed and the Cochrane Library for articles published between January 2015 and July 2025. The search combined MeSH terms and free-text keywords related to AI (Artificial Intelligence, Machine Learning, Deep Learning, AI, Natural Language Processing), osteoporosis (Osteoporosis, Bone Density), and chronic inflammatory rheumatic diseases (Rheumatoid Arthritis, Spondyloarthritis, Ankylosing Spondylitis, Psoriatic Arthritis), as well as clinical concepts (Diagnosis, Prediction, Decision Support, Clinical Decision Support). The PubMed search yielded 1419 records (28 July 2025). Titles and abstracts were screened for relevance, resulting in the exclusion of 1096 articles (55 unrelated to osteoporosis or chronic inflammatory rheumatic diseases, 840 unrelated to AI, 201 unrelated to both). A total of 323 articles were retained for full-text review. The Cochrane Library search identified 3 additional records, of which 1 was added and 2 overlapped with PubMed. All study types were eligible. Articles were included if they addressed the use of AI in osteoporosis or chronic inflammatory rheumatic diseases. No protocol was registered and no formal risk of bias assessment was performed, given the narrative nature of the review and the heterogeneity of included study designs. However, methodology, data sources, model validation strategies, and reported clinical relevance were assessed qualitatively during data extraction and synthesis.

### 2.2. Applications of AI in Rheumatology: An Overview

Applications of AI in rheumatology are mainly based on different machine learning (ML) approaches: supervised, unsupervised, semi-supervised, and reinforcement learning [1,2]. Supervised learning, the most used in medicine, predicts a known clinical outcome (e.g., treatment response) from input data such as imaging or biomarkers. Unsupervised learning, by contrast, does not rely on predefined labels but explores data to group patients into clusters with similar characteristics, thereby identifying subpopulations with comparable risk profiles. Reinforcement learning is based on iterative decision-making guided by rewards and penalties; in rheumatology, a potential application could be dose adjustment according to clinical response, with favorable outcomes reinforcing a decision and poor responses prompting correction [1]. ML techniques encompass both traditional statistical models (e.g., linear or logistic regression, random forests, support vector machines (SVMs)) and more complex approaches such as artificial neural networks. Among these, deep learning—and, in particular, convolutional neural networks (CNNs)—has demonstrated strong potential in medical imaging by automatically extracting discriminative features from complex data [1,2,3]. AI in rheumatology relies on diverse data sources, including imaging, electronic medical records (EMRs), biomarkers, and data from wearable devices. In imaging, radiomics enables the extraction of quantitative features (e.g., shape, texture) before ML analysis. In EMRs, natural language processing (NLP), first applied in axial SpA [4], as well as large language models (LLMs), facilitate automated categorization of unstructured clinical notes and support the systematic exploitation of medical corpora and scientific literature [4].

## 3. AI in the Management of Osteoporosis

### 3.1. Detection of Osteoporosis and Fracture

#### 3.1.1. Reference Standards for the Diagnosis of Osteoporosis

Studies exploring the use of AI for the diagnosis of osteoporosis relied on different reference standards to define the disease. Most applied the World Health Organization thresholds, where osteoporosis is characterized by a T-score—defined as the number of standard deviations from the mean bone mineral density (BMD), assessed in g/cm^2^ by dual-energy X-ray absorptiometry (DXA) in a healthy young adult population—less than or equal to −2.5. Osteopenia, defined by a T-score between −1 and −2.5, was sometimes investigated. A few studies used non-standard criteria, such as clinical features (e.g., osteoporosis: yes/no) and/or biochemical bone turnover markers, while others did not specify their reference standard [1,2,5].

#### 3.1.2. BMD Estimation and Classification from Dedicated Imaging

Recent studies have explored a wide range of AI methods applied to DXA [6,7,8,9,10,11,12,13,14], radiography [6,7,8,9,15,16,17,18,19,20,21,22,23,24,25,26,27,28,29], CT [6,7,16,30,31,32,33,34,35,36,37,38,39,40], MRI [15,41], ultrasound radio frequency [42,43] or kidney–ureter–bladder radiographs [44] to improve the detection and diagnosis of osteoporosis [6,7,15,45,46,47]. These include conventional and enhanced CNNs (U-net [9,10,48], multichannel CNNs [17,33,43], attention-based architectures [30], channel boosting [6], vision transformer–CNNs [49]), as well as hybrid approaches combining transfer learning [6,38], radiogrammetry [22,23], and radiomics [38,50]. Other works focus on 2D and 3D texture and segmentation analysis (segmentation cascades [7,36,37,51], Hounsfield unit (HU) analysis [52], texture analysis [36,52]), sometimes enriched with gradient maps [17,35] or multi-feature fusion [10]. In addition, some methods integrate biomechanical models (FE analyses [53], phantom-patients [23,31]) to enhance robustness. Collectively, these techniques aim to increase the accuracy, precision, sensitivity, and specificity of automated screening, while reducing computational time. Imaging examinations included X-rays of the chest [16,21], lumbar spine [15,54], cervical spine [55], hand and wrist [22,23,24,25,26,27], hip [29], knee [28,56], foot or ankle [57]; CT scans of the chest [16,52]—including low-dose chest CT [49,58] and non-contrast chest CT [59], as well as abdomen-pelvis CT [36,50,52], hand CT [34], and spectral CT [60]; and MRI of the lumbar spine [15] and femoral region [41]. In addition, deep learning models have been developed to enhance the interpretation of DXA scans by enabling the simultaneous assessment of aortic calcifications [61], scoliosis, vertebral degeneration [62] and mortality risk [19]. Risk prediction models based on DXA have also been developed to estimate the risk of diabetes [63] and to predict the failure of cementless arthroplasty in case of osteoporosis [64]. Other models are able to derive pQCT-like spatial density measures from DXA images [65] or conversely, to simulate DXA from CT scans [51]. Another ML model has observed an effective correlation between total radius BMD with the hip and lumbar BMD [66].

#### 3.1.3. BMD Estimation and Classification from Opportunistic Imaging

Radiographs have been widely explored for opportunistic osteoporosis screening, including thoracic and abdominal radiographs [67,68,69,70,71], lumbar spine radiographs [68,69,72,73], hand radiographs [67,74,75], knee radiographs [56,67], foot and ankle radiographs [57], hip and pelvis radiographs [67,76,77], chest radiographs [67,78,79,80], and cervical spine radiographs [55]. Dental imaging has also been evaluated for opportunistic detection, using either panoramic radiographs [81,82,83,84,85,86,87,88,89,90,91,92] or cone beam CT [86,93,94,95]. BMD has further been estimated in coronary artery calcium scans [96]. Opportunistic CT has demonstrated feasibility across multiple locations and contexts: lumbar spine CT [68,97,98,99,100,101,102,103,104,105,106,107,108,109,110,111,112,113], chest CT [97,114,115,116,117,118,119,120,121,122,123,124,125,126,127]—including non-contrast [128], and low dose acquisitions [125,129,130,131,132,133], abdominal CT [97,102,119,120,134,135,136,137,138]—including low-dose [139] and MDCT [136], as well as whole body CT [140], shoulder CT [141], radius CT [142], tibia CT [142], and ultra-low dose hip CT [143]. Applications have been reported in lung cancer screening [114,129], chronic obstructive pulmonary disease (COPD) follow-up [115], preoperative thoracolumbar CT for spine surgery [117], and in patients undergoing spine surgery [106] or transcatheter aortic valve replacement [123]. Notably, a study observed that opportunistic osteoporosis screening on vertebral CT scans may provide interpretations comparable to dedicated scans [110]. Another study showed that low-dose CT provides predictive performance comparable to standard-dose CT; however, variations in tube voltage markedly reduce radiomic feature reproducibility and model efficacy, highlighting the necessity of consistent acquisition parameters [125]. Then, a study build a ML framework to identify which vertebrae would be excluded from DXA analysis based on the CT attenuation of the vertebrae [113]. Chest CT has also been association with bone turnover markers to predict osteoporosis [118], while abdominal CT has been used to assess psoas muscle morphology for predicting osteoporosis [135]. Lumbar MRI has similarly been investigated for osteoporosis prediction [103]. CT-based measures have shown superiority over DXA in identifying patients with reduced bone mass who sustained vertebral fractures [98]. Osteoporosis has also been predicted when lumbar fractures were observed on MRI [144] or CT [145]. As with dedicated imaging, various computational approaches have been evaluated, including integrated multimodal radiomics [68,101,104,107,108,118,121,134,139,144], machine vision [146], U-net [71], feature-based broad learning system [72], data augmentation strategies (FAST [74]), HU analysis [115,117,121], automatic segmentation [139,140], bone morphometry [121], multi-feature DCNN model [122], phantomless internal calibration [130]. ML applied to X-rays has additionally classified trabecular bone structure in osteoporotic patients [146].

#### 3.1.4. Opportunistic Fracture Screening

Model for opportunistic fracture screening have also been developed across imaging and modalities: on radiographs [73,147,148,149,150], CT [112,151,152,153,154,155,156,157], and MRI [156]. These models were designed primarily for the screening of vertebral fracture [73,112,147,148,149,151,152,153,154,155,157], but also for hip fracture [150]. This opportunistic screening has relied on a variety of computational approaches, such as radiomics [151], neuronal network-based automatic spine segmentation and volumetric BMD extraction [154], and texture analysis [155]. For vertebral fractures, some studies have used variations of Genant’s semiquantitative vertebral fracture classification [148].

### 3.2. Prediction of Osteoporosis and Fracture

Beyond imaging, a wide range of approaches have been investigated to predict osteoporosis [90,145,158,159,160,161,162,163,164,165,166,167,168,169,170,171,172,173,174,175,176,177,178,179,180,181,182,183,184,185,186,187,188,189,190,191,192,193,194,195,196,197,198], initial fracture risk [23,73,199,200,201,202,203,204,205,206,207,208,209,210,211,212,213,214], subsequent one [213,215,216] or bone loss rates [217,218]. Most studies focused on predicting initial fracture risk in non-osteoporotic patients, although some limited the analysis to osteoporotic populations [213]. Models based on clinical data and EMRs [158,159,160,161,162,163,164,165,166,167,168,169,170,171,172,173,174,175,176,177,201,202,212] have been developed, sometimes combined with DXA [178,203,204,218], radiographs [179,180,181]—including panoramic radiographs [90]—CT scans [182,183,198,200,204], or ultrasounds [184]. Imaging has also been applied for prediction without incorporating clinical variables, using DXA [73] with the bone strain index [211] and CT [199,200,210,214]. Other studies have relied on analyses of bone turnover markers [118,185,186,187]. Non-traditional biomarkers have also been explored, such as fecal pH [188], heavy metals [189], radiofrequencies [190] or electromagnetic waves [191]. Across these diverse modalities, the common outcome was the prediction of osteoporosis or low bone mass risk, rather than direct BMD estimation or fracture prediction. Factors associated with the trabecular bone score have also been investigated [192]. In parallel, a study compared the performance of FRAX with and without DXA, demonstrating that AI-augmented models without DXA achieved a similar level of efficacy to predict fracture risk [193]. Further research has examined clinical or EMR-derived risk factors associated with longitudinal declines in bone density [219], or identified key immune genes in genetic studies linked to osteoporosis [220,221,222,223]. In addition, ML models have been applied to predict sleep quality in patients with osteoporosis [224]. NLP has also been used to analyze orthopantomogram radiographic notes in order to predict bone loss [225].

### 3.3. Specific Populations and Clinical Contexts

AI models for osteoporosis have been applied across diverse populations. These include patients with specific comorbidities, such as postmenopausal women with diabetes [162,175,205,206,226], patients on dialysis [194], those with hyperparathyroidism [195], high cardiovascular risk [196], or thyroid cancer with TSH suppression [142]. They have also been tested in post-gastrectomy cohorts [176], in patients undergoing spine surgery [106,117,227], and in patients with RA [228,229]. Beyond these disease contexts, applications have extended to particular demographic or physiological groups, such as athletes [177], children [24,230], patients below the recommended ages for DXA screening [169], elderly men [173,207,231], and women living at high altitude [197].

### 3.4. Screening Strategies

Beyond opportunistic detection of osteoporosis and fractures on imaging, recent advances in AI have integrated NLP techniques to enhance opportunistic screening strategies, enabling the automated extraction of BMD values from DXA reports to identify individuals at risk of osteoporosis [232]. In parallel, self-assessment tools based on ML have been designated to facilitate early osteoporosis detection outside clinical settings. Such model have demonstrated the potential to improve population-level screening coverage and to support preventive strategies in at-risk groups [233].

### 3.5. Therapeutic Monitoring

Clinical decision support systems (CDSS) are increasingly explored to optimize treatment strategies and monitor therapeutic outcomes in osteoporosis. Several models integrate DXA results, clinical history, and laboratory or diagnostic data to generate individualized therapeutic recommendations [234]. Other AI approaches aim to predict drug interactions [235], treatment efficacy [236], or the risk of osteonecrosis of the jaw [237], and to estimate the BMD response following osteoporosis therapy [238].

In a recent study, ChatGPT-4.0-generated treatment recommendations were systematically compared with those of five experienced clinicians after an osteoporosis diagnosis, revealing a high level of concordance [239]. Beyond algorithmic prediction, several digital tools have been developed to assist clinical decision-making and patient management. These include CDSSs integrated within EMRs with automated alerts, the Osteoporosis Advisor (OPAD) providing guideline-based recommendations, and national Fracture Liaison Service databases (FLS-DBs) used to monitor quality of care and secondary prevention outcomes. A recent example is the construction of a novel online calculator for predicting osteoporosis risk in Chinese patients with type 2 diabetes, which demonstrates the potential for population-specific digital prediction tools [175]. Complementary mobile and digital health solutions—such as FRAX App, My Osteoporosis Journey, My Osteoporosis Manager—support risk calculation, education, and adherence monitoring. Finally, e-Triage systems, telemedicine platforms, and automated reminder tools have been implemented to streamline referrals, improve follow-up, and strengthen long-term patient engagement [240].

A summary of AI applications in osteoporosis, including data sources, AI models, validation strategies, and clinical relevance, is provided in Table 1.

## 4. AI in the Management of Chronic Inflammatory Rheumatic Diseases

### 4.1. Early Diagnosis Using Electronic Medical Records and Claims Data

ML models using EMRs and administrative claims data have demonstrated promising results for the early identification of axial SpA and PsA [241,242,243,244,245]. Several studies reported that AI-based approaches outperformed traditional clinical models in diagnostic accuracy and timeliness [242,246,247]. In psoriasis, a model based on blood sample showed a efficacy to distinguish PsA from cutaneous psoriasis [248]. Similarly, in SpA, ML methods have been applied to predict HLA-B27 status using clinical and laboratory data [249].

### 4.2. Imaging-Based Diagnosis of Sacroiliitis and Inflammation in Axial SpA

AI has been extensively explored for the diagnosis and characterization of axial SpA particularly through imaging-based analysis [250]. AI systems have shown expert-level performance for the detection and classification of sacroiliitis on both radiographs [251,252,253,254,255], and MRI [245,256,257,258,259,260,261,262,263,264,265,266,267,268], with advanced CNN and Inception-based architectures improving sensitivity [269]. Automated sacroiliac joint segmentation pipelines [270,271,272] highlight the potential for standardization across cohorts. MRI-based models have also been developed to quantify bone marrow edema [273,274], erosions [275], osteitis [275], ankylosis, and hip involvement [276], as well as to differentiate inflammatory from degenerative changes [277]. Recent works have also explored CT translation models from MRI [278] and multimodal approaches integrating imaging and clinical variables to enhance diagnostic accuracy [245,270].

Beyond sacroiliitis assessment, AI-driven imaging analysis has expanded to other inflammatory rheumatic diseases. Models using hand MRI have been trained to detect and interpret inflammatory lesions in RA, peripheral SpA, and PsA [279]. Automated MRI-based classification of spondylitis has also been reported [280]. In addition, AI models have been applied to screen osteoporosis risk in RA patients using the second metacarpal cortical index [229].

### 4.3. Prediction of Disease Progression

AI and ML approaches have shown growing potential to predict radiographic and structural progression across inflammatory rheumatic diseases. In axSpA, ML models have outperformed traditional statistical methods for predicting radiographic progression [281,282], while integrated pipelines combining imaging data and longitudinal clinical follow-up have improved individualized risk stratification [283]. In PsA, deep learning models have been used to characterize disease heterogeneity, distinguish clinical phenotypes [284], and identify variables associated with minimal disease activity [285]. Similarly, in RA, AI-based models leveraging radiographs, MRI, and ultrasound data have been used to predict radiographic progression, particularly through automated scoring systems and radiomic feature extraction [4,286]. More recently, dynamic ML architectures have been developed to forecast future disease activity scores in RA [287,288].

### 4.4. Prediction of Therapeutic Response

A recent systematic review identified 89 studies evaluating various AI methodologies for prediction of therapeutic response in RA and SpA, most of which relied on supervised learning approaches, particularly random forests, SVMs, and neural networks. Reported performances were generally good, with areas under the ROC curve (AUCs) ranging from 0.63 to 0.92, depending on the datasets and analytical methods used [4]. In RA, predictive features have included clinical indices (DAS28, CDAI), biomarkers (CRP, ACPA), imaging findings (MRI, ultrasound), and multi-omics signatures. AI models have been applied to predict response to methotrexate, TNF inhibitors, IL-6 inhibitors, JAK inhibitors, abatacept, and rituximab, enabling personalized treatment optimization [286,289]. Deep learning–based clustering approaches have further identified distinct patient subgroups in RA that respond differently to biologic or targeted synthetic DMARDs, underscoring the potential of AI for treatment stratification and precision medicine [290]. Recent work has also explored the ability of ML models to predict sustained biologic or targeted synthetic DMARD-free remission in RA patients, identifying clinical and laboratory factors associated with successful treatment discontinuation [291]. In SpA, particularly ankylosing spondylitis, neural network–based models have outperformed conventional statistics in predicting response to TNF-α inhibitors, identifying CRP and ESR as consistent key predictors [286,289,292]. Other ML models integrating demographic, laboratory, and disease-related variables have successfully forecasted improvements in BASDAI and BASFI scores and identified factors associated with treatment failure [250]. In PsA, similar frameworks have been developed to predict response to biologic DMARDs (bDMARDs) and to model treatment outcomes using clinical and imaging-derived features [289,293,294].

### 4.5. Prediction of Extra-Articular and Systemic Complications

Beyond articular manifestations, AI applications have expanded to the prediction of extra-articular and systemic complications linked to inflammatory rheumatic diseases. In SpA, ML models have been developed to predict anterior uveitis [295], myocardial infarction, and cardiovascular risk [296,297]. In RA, predictive algorithms have been designed to assess cardiovascular events, treatment-related adverse effects, and even osteoporosis risk in long-term follow-up cohorts [229,235]. Moreover, recent studies have incorporated psychological and social variables to predict the differential diagnosis of rheumatic and musculoskeletal diseases, highlighting the emerging role of AI in capturing the biopsychosocial dimensions of disease expression and progression [298].

### 4.6. Therapeutic Monitoring and Digital Self-Management

AI and digital health technologies are increasingly being integrated into the monitoring and self-management of inflammatory arthritis. Regarding therapeutic monitoring, in axial SpA, patient-initiated telemedicine consultations have been shown to reduce the frequency of in-person visits without compromising clinical outcomes in patients with stable disease [299]. Likewise, ML applied to activity tracker data accurately predicted flares in RA and axial SpA [300]. Concerning digital self-management, proof-of-concept studies in axSpA demonstrated the feasibility of smartphone-based self-monitoring, which enhances patient engagement and supports tight control strategies [286]. In RA, CNNs have been applied for dorsal finger fold recognition to detect and monitor joint swelling, offering a non-invasive digital biomarker for disease activity assessment [301]. In PsA, the Psorcast project employs a smartphone application to capture digital symptom data and patient-reported outcomes (PROs), using AI algorithms to predict treatment response and flare–remission cycles [302]. Similarly, the IMI2 IDEA-FAST project leverages wearable and mobile technologies to identify digital biomarkers of fatigue, sleep disturbances, and daily functioning, supported by advanced analytical frameworks [302]. Moreover, the iPROLEPSIS program uses explainable AI and predictive analytics to track the transition from psoriasis to PsA and to tailor prevention and treatment strategies. Its digital suite includes the miPROLEPSIS app for risk monitoring, an AI-based lifestyle coach, and tools addressing sleep, stress, and pain management [302]. Beyond these applications, reinforcement learning has been proposed as a future approach to enable autonomous, adaptive therapeutic decision-making in rheumatology [303].

As for osteoporosis, a summary of AI applications in chronic inflammatory rheumatic diseases, is provided in Table 2.

## 5. Challenges, Limitations, and Ethical Considerations

### 5.1. Data Quality

The reliability of AI models in rheumatology is closely tied to the quality and representativeness of the datasets used for training. Current studies reveal substantial heterogeneity in data sources, ranging from EMRs and administrative claims to imaging repositories and publicly available datasets of uncertain provenance and variable data quality [304]. EMRs provide rich clinical detail but suffer from inconsistencies in coding and documentation, whereas claims databases capture larger populations but lack clinical granularity, as they are primarily designed for billing rather than research purposes and often omit essential clinical details such as laboratory results, disease activity scores, or imaging data, with diagnostic coding that may be imprecise or incomplete. Furthermore, the absence of universally accepted gold standards—whether DXA-derived T-scores, volumetric BMD from QCT, or clinical fracture outcomes—complicates cross-study comparisons and contributes to variability in reported results [305].

### 5.2. Model Explainability

A wide variety of AI architectures have been applied in rheumatology and bone imaging, reflecting both the dynamism and fragmentation of the field [163,306]. CNNs remain the backbone of most image-based analyses due to their ability to automatically extract spatial features [307,308]. Enhanced architectures—such as U-net, attention-based CNNs, and vision transformer, CNN hybrids, further improve segmentation accuracy and feature localization, particularly in complex anatomical regions [309]. In parallel, transfer learning and radiomics-based approaches leverage pre-trained networks or handcrafted image descriptors to optimize performance in smaller datasets. Classical ML algorithms, including random forests, SVMs, and regression-based models, are also employed for tabular or multimodal data, offering greater interpretability at the expense of predictive depth. While deep learning models typically achieve higher accuracy, they often lack transparency, whereas simpler algorithms provide clearer insight into decision pathways but may be less powerful for high-dimensional data [310]. Consequently, there is no consensus on which model is most clinically relevant, as performance and interpretability depend heavily on dataset quality, preprocessing pipelines, and target outcomes [311,312,313].

A major challenge is therefore model explainability, understanding how predictions are generated and which variables most influence them. In this context, interpretability techniques such as SHAP (SHapley Additive exPlanations) and LASSO (Least Absolute Shrinkage and Selection Operator) have gained prominence. SHAP, derived from game theory, decomposes a model’s prediction into the contribution of each feature, allowing clinicians to identify which parameters (e.g., bone density, age, inflammatory markers) most drive a given output. LASSO, a penalized regression method, enhances model simplicity and transparency by shrinking or eliminating variables that contribute little to prediction accuracy. Together, these techniques enable researchers to highlight key determinants of model behavior, reduce overfitting, and build trust in AI-assisted decision tools.

### 5.3. Model Validation

Although many studies report high performance metrics, these are often based on internal validation within a single dataset, raising concerns of overfitting and limited applicability in real-world practice. External validation, ideally multicenter, remains rare, despite being essential for generalizability [21,77,78,152,229,314]. In practice, only a minority of studies report comparison with standard clinical tools. Most AI models therefore remain “high-performing” only within the data on which they were trained, making their reliability in real-world conditions largely unknown. To promote methodological transparency and reproducibility, structured reporting frameworks such as TRIPOD (Transparent Reporting of a multivariable prediction model for Individual Prognosis Or Diagnosis) have been developed [315]. The TRIPOD Statement, first published in Annals of Internal Medicine in 2015, provides detailed guidance on how to report the development, validation, and performance of clinical prediction models. It includes 22 mandatory items covering study design, data handling, model specification, validation procedures, and performance metrics such as calibration and discrimination. Extensions such as TRIPOD-AI and TRIPOD-ML now adapt these principles to modern ML approaches [316,317]. Beyond TRIPOD, several complementary frameworks have been developed to enhance the transparency, reproducibility, and methodological quality of AI-based prediction studies, e.g., PROBAST evaluates the risk of bias and applicability, while CHARMS provides guidance for critical appraisal in systematic reviews. CLAIM focuses on reporting standards for AI in medical imaging, and CONSORT-AI and SPIRIT-AI extend clinical trial reporting to interventions involving AI. More recent initiatives, such as FUTURE-AI, REFORMS, and PROBAST-AI, further emphasize fairness, accountability, and ethical integrity in the development and validation of AI-driven clinical models. However, despite the availability of these robust methodological frameworks, only a minority of studies provided sufficient information to enable full evaluation according to TRIPOD, PROBAST, or CLAIM criteria. Key elements such as external validation, standard references, handling of missing data, reproducibility, or transparent reporting of model development were often missing.

### 5.4. Clinical Relevance

Overall, the current literature provides far more evidence of technical feasibility than of clinically reliable or externally validated performance. Many models are developed using retrospective data, evaluated against surrogate endpoints, and tested in ideal, controlled conditions that do not reflect the heterogeneity of real-world practice. The clinical relevance of AI models proposed in rheumatology remains debated. For instance, one study involving more than 150 men reported no reduction in fracture incidence following AI-assisted opportunistic screening—largely because patients were not treated after diagnosis [231]. Conversely, other investigations have demonstrated clearer clinical utility. In primary care, AI algorithms applied to EMRs can suggest vertebral fracture assessment (VFA) or DXA testing, or even support treatment initiation [171]. In underserved areas, certain models can estimate osteoporosis risk without DXA, thereby improving access to diagnostic assessment [193]. In medical imaging, AI approaches have also been used to reconstruct CT-equivalent images from MRI, which may reduce radiation exposure, limit redundant examinations, and shorten diagnostic delays [278]. Nevertheless, the clinical pertinence of AI tools remains limited by several major obstacles. Selecting the most advanced and reliable model is often arbitrary given the abundance of available architectures—ranging from conventional CNNs and U-Net derivatives to attention-based networks, hybrid radiomic fusion models, and transformer-based systems. Moreover, a substantial gap persists between computational development (proof-of-concept studies and statistical performance) and actual clinical implementation. Even when an AI model demonstrates high performance and meets established quality-control criteria, transitioning from AI model development to real-world clinical deployment remains highly challenging. To date, only a small number of AI-based tools are commercially available or integrated into routine practice. Beyond model development, researchers and companies must design robust software solutions that can be integrated into the heterogeneous and often incompatible healthcare information systems used across institutions. This requires substantial technical adaptation, adequate computational capacity, and reliable integration pathways to avoid generating excessive data flow or disrupting clinical workflows. Legal and regulatory barriers further complicate adoption, particularly regarding data protection, cybersecurity, and the risks associated with transmitting clinical information through external software platforms. In parallel, administrative and regulatory bodies must evaluate, certify, and authorize an increasing number of heterogeneous AI tools. These combined technical, legal, and administrative constraints considerably limit the real-world implementation of AI in rheumatology despite promising early results.

### 5.5. Acceptability by Practitioners and Patients

Beyond technical performance, the integration of AI into clinical rheumatology depends on its acceptability among clinicians and patients. Practitioners remain cautious toward opaque systems, while patients may question the role of automated decision-making in sensitive diagnostic or therapeutic contexts [318]. The perceived reliability, ease of workflow integration, and ability to complement clinical expertise are key to adoption. In this context, conversational AI tools such as ChatGPT are increasingly explored by healthcare professionals for literature synthesis, education, and decision support, underscoring both their potential and the need for rigorous validation of generative models [319].

Importantly, one of the major limitations of most existing AI models is the lack of explainability. Current approaches rarely provide clinically meaningful insights into why a model produces a given prediction. This opacity significantly hinders clinician trust and remains a major barrier to routine use, in addition to the technical, ethical, and legal considerations discussed elsewhere in the manuscript. Recent studies examining clinician acceptability and trust in AI similarly highlight explainability as a central determinant of adoption, with healthcare professionals expressing the need for transparent, interpretable outputs before integrating AI-driven recommendations into their practice.

### 5.6. Ethical and Legal Considerations

Ethical and regulatory challenges remain central to AI deployment in rheumatology. Ensuring algorithmic fairness and mitigating demographic bias are essential to avoid reinforcing existing health disparities. Data protection frameworks such as GDPR (Europe) and HIPAA (United States) impose strict rules on consent and data use, yet cross-jurisdictional variability hampers multicenter research. Furthermore, legal accountability in cases of erroneous predictions remains undefined, raising questions regarding the respective liabilities of developers and clinicians. Future integration must therefore rest on robust ethical and legal frameworks that guarantee transparency, accountability, and equitable access.

### 5.7. Cost-Effectiveness

Economic evaluations suggest that AI-assisted screening and decision tools can be cost-effective. In osteoporosis and cardiovascular disease, AI-based models generated incremental benefits of $227 per male and $65 per female patient compared with current strategies [320]. In Germany, AI-driven opportunistic osteoporosis screening achieved an estimated cost of €13,340 per QALY gained—well below the conventional threshold of €60,000 [321]. These findings support the potential of AI integration to enhance both clinical outcomes and healthcare system efficiency.

## 6. Discussion

Recent literature confirms that AI is rapidly advancing in rheumatology, particularly in medical imaging and through the valorization of routine clinical data, leading to potential time and cost savings [322]. In bone research, AI models have been applied to estimate BMD, diagnose osteoporosis, and predict or detect fractures and mortality using opportunistic or dedicated imaging, routine EMRs, and more innovative inputs such as ultrasounds, multi-omics profiles, and biomarker [323]. Hybrid strategies integrating imaging, clinical, and biological information are increasingly emerging. Importantly, several models have been adapted to specific populations—such as diabetic, dialysis, post-gastrectomy, pediatric, or athletic cohorts—highlighting their potential generalizability to atypical or high-risk groups. NLP has also enabled automated screening for osteoporosis within EMRs, while other models predict longitudinal outcomes such as bone density loss, treatment response, or the occurrence of adverse events. In inflammatory rheumatic diseases, AI tools have been developed to assist diagnosis and disease activity monitoring from both imaging and EMR data. In axial SpA, AI-based imaging algorithms have shown efficacy in differentiating inflammatory sacroiliitis from degenerative changes and in detecting structural lesions such as erosions, ankylosis, and bone marrow edema on radiographs or MRI. Moreover, predictive models can now estimate disease activity or therapeutic response according to patient subgroups and treatment classes, opening new perspectives for precision medicine in rheumatology.

Nevertheless, significant challenges persist before these tools can be reliably implemented in clinical practice. Data quality remains highly variable. Heterogeneity in reference standards limits cross-study comparability. The proliferation of AI architectures also complicates interpretability. Model overfitting remains frequent, with studies reporting excellent internal validation but limited external testing. Systematic adoption of explainability frameworks such as SHAP and LASSO is therefore essential to clarify feature contributions and improve transparency. External validation and standards such as TRIPOD are crucial to ensure reproducibility and generalizability. Clinical applicability is further constrained by limited clinician and patient acceptability. AI systems are often perceived as “black boxes,” raising concerns about their reliability and translational validity in real-world clinical settings. Ethical and regulatory concerns—including data privacy, model transparency, and medico-legal responsibility in the case of erroneous predictions—must also be addressed. Despite these limitations, emerging evidence suggests that AI-assisted approaches may offer meaningful cost-effectiveness benefits by optimizing workflows and improving diagnostic efficiency.

In perspective, AI applications in osteoporosis and inflammatory rheumatic diseases appear to be shifting from model development toward real-world clinical integration. The priority is no longer to maximize AUC values, but rather to strengthen robustness, external validity, clinical utility and implementation, and cost-effectiveness. Several important questions remain only partially addressed, including how to reliably demonstrate real-world robustness and clinical usefulness of AI models, and how to integrate underexplored data modalities such as wearable-derived signals, multi-omics datasets, and PROs collected through mobile platforms. In addition, very few studies have investigated patient and clinician acceptability of AI tools, even though these aspects are critical for successful adoption in routine practice.

## Figures and Tables

**Table 1:** Overview of AI applications in osteoporosis: data sources, AI models, validation and clinical relevance. Detailed references are reported in the corresponding sections of the text.

Clinical Application	Data Source	AI Models	Reference Standard/Validation	Clinical Relevance
Diagnosis/BMD estimation (dedicated imaging)	DXA, X-ray, CT, MRI, Ultrasound RF, kidney–ureter–bladder radiographs	CNN, (U-Net, multichannel CNN, attention-based architectures, vision transformer–CNNs), transfer learning, radiogrammetry, radiomics, 2D/3D texture and segmentation analysis, Hounsfield unit-based models, gradient maps, multi-feature fusion	WHO T-score (≤−2.5) (most studies); clinical diagnosis; bone turnover markers	Automated BMD estimation; improved diagnostic accuracy and enhanced screening efficiency
Opportunistic osteoporosis screening	Radiographs, CT (low-dose, non-contrast), dental imaging, MRI,	Multimodal radiomics, machine vision, U-net, feature-based broad learning system, data augmentation strategies, Hounsfield unit analysis, automatic segmentation, bone morphometry, multi-feature DCNN model, phantomless internal calibration	WHO T-score (≤−2.5); clinical diagnosis; bone turnover markers	Opportunistic identification of osteoporosis without additional imaging, enabling low-cost and scalable screening
Opportunistic fracture detection	X-ray, CT, MRI	CNN, radiomics, texture analysis	Genant semiquantitative classification; expert annotation	Automatic detection of acute vertebral and hip fractures
Fracture and osteoporosis risk prediction	Clinical data and EMRs, sometimes combined with DXA, radiographs, CT or US; DXA; CT; bone turnover markers; non-traditional biomarkers (fecal pH; heavy metals, RF; electromagnetic waves)	ML models, artificial neural networks, ensemble models, support vector machines	WHO T-score (≤−2.5); clinical diagnosis; bone turnover markers; incident fracture	Personalized fracture risk stratification; treatment decision-making support;
Therapeutic monitoring/decision support	DXA, clinical data, laboratory data	ML-based clinical decision support systems (CDSS)	Concordance with clinicians; treatment response	Personalized therapy optimization; drug interaction risk assessment; treatment efficacy prediction.

**Table 2:** Overview of AI applications in chronic inflammatory rheumatic diseases: data sources, AI models, validation, and clinical relevance. Detailed references are reported in the corresponding sections of the text.

Clinical Application	Data Source	AI Models	Reference Standard/Validation	Clinical Relevance
AI-assisted diagnosis (EMR-based)	Electronic medical records, administrative claims, laboratory data, blood samples	ML classifiers (random forest, SVM, neural networks), ensemble models	Expert clinical diagnosis; classification criteria; laboratory markers	Early identification of axSpA and PsA; reduced diagnostic delay; clinical decision support for physicians
Imaging-based diagnosis of sacroiliitis (axSpA)	Radiographs, MRI, CT	CNNs (Inception-based, attention-based), automated segmentation pipelines, multimodal models	Expert radiologist annotation; ASAS criteria; MRI inflammation scores	Standardized and accurate detection of sacroiliitis; expert-level performance; improved diagnostic consistency
Imaging-based assessment of inflammatory lesions	MRI	CNNs, segmentation models, radiomics, texture analysis	Expert annotation; validated imaging scores	Quantification of inflammatory burden; differentiation of inflammatory vs. degenerative changes
Prediction of radiographic and disease progression	Radiographs, MRI, ultrasound, longitudinal clinical data	ML models, deep learning, dynamic prediction architectures	Radiographic progression scores; disease activity indices	Individualized risk stratification; anticipation of structural damage
Prediction of therapeutic response	Clinical indices, biomarkers, imaging data, multi-omics data	Supervised ML (random forest, SVM), neural networks, deep clustering	Treatment response criteria; AUC-based performance metrics	Personalized treatment optimization; identification of responders and non-responders
Prediction of remission and treatment discontinuation	Clinical and laboratory follow-up data	ML models, deep learning clustering	Sustained remission definitions; clinical expert validation	Support for treatment tapering and drug-free remission strategies
Prediction of extra-articular and systemic complications	Clinical data, EMR, laboratory variables	ML models, ensemble learning	Clinical diagnosis of complications	Early detection of comorbidities; improved long-term risk management
Therapeutic monitoring and digital self-management	Wearables, activity trackers, smartphones, patient-reported outcomes	ML models, CNNs, explainable AI, reinforcement learning	Clinical outcomes; flare detection; clinician concordance	Remote monitoring; flare prediction; enhanced patient engagement and self-management

## Data Availability

The original contributions presented in this study are included in the article. Further inquiries can be directed to the corresponding author.
